# Bradycardia in Patients with Subcutaneous Implantable Defibrillators—An Overestimated Problem? Experience from a Large Tertiary Centre and a Review of the Literature

**DOI:** 10.31083/j.rcm2310352

**Published:** 2022-10-18

**Authors:** Kevin Willy, Florian Doldi, Florian Reinke, Benjamin Rath, Julian Wolfes, Felix K. Wegner, Patrick Leitz, Christian Ellermann, Philipp Sebastian Lange, Julia Köbe, Gerrit Frommeyer, Lars Eckardt

**Affiliations:** ^1^Department for Cardiology II: Electrophysiology, University Hospital Münster, 48149 Münster, Germany

**Keywords:** S-ICD, bradycardia, complications

## Abstract

**Background:**

The subcutaneous ICD (S-ICD) has developed as a valuable alternative to transvenous implantable cardioverter defibrillator (ICD) systems. However there are certain peculiarities which are immanent to the S-ICD and may limit its use. Besides oversensing the main issue is the missing option for antibradycardia pacing. To evaluate the actual need for pacing during follow-up and changes to transvenous ICD we analyzed our large tertiary centre registry and compared it with data from other large cohorts and trials.

**Methods and Results:**

We found out that in the 398 patients from our centre, there was a need for changing to a transvenous ICD in only 2 patients (0.5%) during a follow-up duration of almost 3 years. This rate was comparable to data obtained from other large data sets so that in the pooled analysis of almost 4000 patients the rate of bradycardia-associated complications was only 0.3%.

**Conclusions:**

The use of the S-ICD is safe in a variety of heart diseases and the need for antibradycardia stimulation is a very rare complication throughout many different large studies. Clinicians may take these results into account when opting for a certain ICD system and the S-ICD may be chosen more often also in elderly patients, in whom the risk for bradycardia is deemed higher.

## 1. Introduction

Since its implementation, the implantable cardioverter defibrillator (ICD) has been shown to be a safe and effective treatment option for patients at high risk for life threatening arrhythmias. Despite its pivotal role in the prevention of sudden cardiac death, ICD use is associated with short- and long-term complications leading to a significantly increased morbidity and mortality. Implanted defibrillator leads are, e.g., vulnerable to fractures leading to inappropriate therapy and infections [1, 2]. Hence, the subcutaneous ICD (S-ICD) was developed to reduce lead-related complications and infectious risk with accompanying demanding and risky lead extraction surgery [2, 3]. As the S-ICD on the other hand is not able to provide chronic pacing, it is not suitable for patients having or potentially developing a need for bradycardia-related pacing, anti-tachycardia pacing (ATP), or cardiac resynchronization therapy (CRT) [3]. Thus, the best candidates for S-ICD seem to be young patients expected to outlive the transvenous lead life expectancy or with difficult venous access (e.g., congenital venous anomalies) and no potential need for any kind of pacing [3, 4].

The PRAETORIAN trial has shown non-inferiority of the S-ICD compared to the transvenous ICD with regard to device-related complications and inappropriate shocks [5] and current ESC guidelines [6] have given the S-ICD a IIa recommendation in patients not having an indication for cardiac pacing. In addition, according to the AHA/ACC/HRS, the S-ICD has a class I recommendation [7] for patients with an additional high risk for infections or without adequate venous access. As both devices seem similar with regard to complications and efficacy [3, 8], it remains difficult for physicians to assess who will require bradycardia-related pacing or ATP during follow-up. Therefore, we aimed at providing experiences and data on this topic from a large tertiary centre and presenting a detailed review of literature discussing the problem of adequate device selection in patients at risk of sudden death.

## 2. Prospective Registry Data

For analysis of bradycardia-associated complications and changes to transvenous ICDs either due to bradycardia or for cardiac resynchronization therapy we analyzed our prospective large single center registry. As the data were only used in a retrospective and anonymous manner a statement of the ethics committee was not necessary and therefore not obtained. For the same reason there was no informed consent obtained. As the trial was of retrospective nature it was also not registered as a clinical trial.

The registry includes 398 consecutive patients who received a subcutaneous ICD for primary or secondary prevention of sudden cardiac death (SCD) between June 2010 and November 2021. The patient cohort had a mean age of 42.4 ± 15.6 years (min 12 years, max 78 years) and a mean LV-EF of 49.7 ± 14.4% at implantation. About 2/3 of patients were male (67.3%) with numerous underlying cardiac diseases (please see Table 1). Approximately half of the S-ICD were implanted for primary (53.5%) and for secondary prevention (46.5%) of which most patient survived an episode of ventricular fibrillation (116/185; 62.7%).

**Table 1.** **Patient baseline characteristics**.

Baseline characteristics		Total (n = 398)
Male (n)		268 (67.3%)
Age (years)		42.4 ± 15.6
Left ventricular ejection fraction (%)		49.7 ± 14.4
Primary prevention (n)		213 (53.5%)
Underlying heart disease (n)		
	Ischemic cardiomyopathy	64 (16.1%)
	Non-ischemic cardiomyopathy	60 (15.1%)
	Ion channelopathy	66 (16.6%)
	Hypertrophic cardiomyopathy	57 (14.3%)
	Idiopathic ventricular fibrillation	49 (12.3%)
	Congenital heart disease	32 (8.0%)
	Valvular heart disease	19 (4.8%)
	Other heart diseases (e.g., myocarditis, mitral valve prolapse syndrome)	49 (12.3%)
ECG parameters before implantation		
	Mean QRS width	106 ± 26
	QRS >120 ms	64 (16.1%)
	LBBB	26 (6.5%)
	RBBB	39 (9.8%)
	AV-Block I + LAHB	24 (6.0%)
	PQ interval	170 ± 32
	PQ >200 ms	38 (9.5%)
	Mean heart rate	68 ± 14
	Patients on betablockers	296 (74.3%)
	Patients on AAD	45 (11.3%)

ECG, Electrocardiogram; LBBB, Left-Bundle Branch Block; RBBB, Right Bundle-Branch Block; AV, Atrioventricular; AAD, Antiarrhythmic Drugs.

Overall, during a mean follow-up of about 3 years (range 2 up to 3504 days, 191 patients with a follow-up duration >2 years (48.0%)) only 2 patients (0.5%) suffered from relevant clinical bradycardia in our large single-center registry. Symptomatic bradycardia with recurrent symptomatic sinus bradycardia and pauses of up to 3s occurred in one woman after surgical resection of a ventricular myxoma, operative reconstruction of the tricuspid valve and implantation of a mitral valve prosthesis in whom the S-ICD was implanted for primary prevention of SCD in the presence of a leftventricular ejection fraction (LV-EF) of 30%. As she received appropriate S-ICD therapy due to monomorphic ventricular tachycardia (VT) possibly accessible to ATP, the ICD system was changed to a transvenous DDD-ICD system one month after implantation. Mortality during follow-up was low. 6 patients (1.5%) died during follow-up, 3 because of infections with septic course, one because of malignoma and one during a cardiothoracic surgery of the aortic root. The case of the last remaining patient is described as follows.

**Fig. 1.**
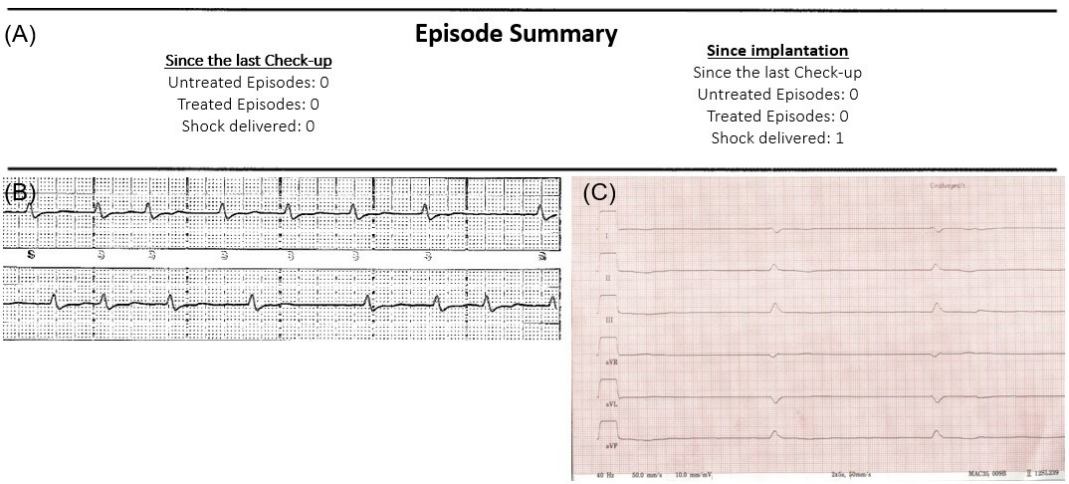
**Case presentation of a patient with ischemic cardiomyopathy being resuscitated due to bradycardia with an implanted S-ICD. **72-year old patient with ischemic cardiomyopathy and implanted S-ICD (A, B) initially presenting to our clinic with shunt-phlegmone and consequently undergoing CPR with pulseless electric activity as primary rhythm. After ROSC the patient showed the presented bradycardia (C). An acute myocardial ischemia was excluded via coronary angiogram. As the patient had suffered substantial hypoxic damage with subsequent multi-organ failure further therapeutic measures were not pursued and the patient died shortly after.

One other 72 years old patient with an ischemic cardiomyopathy and chronic kidney disease who had received the S-ICD for primary prevention of SCD was admitted to our intensive care unit after being hospitalized for shunt infection. In course of the infective situation, the patient was found unconscious in his bed and was then subject to cardiopulmonary resuscitation. His first documented rhythm was a bradycardic ventricular escape rhythm. After initially successful resuscitation the patient persistently suffered from cardiogenic shock so that following the assumed patients’ will and the wishes of the patient’s relatives further intensive care measures were omitted, and the patient died shortly after. Fig. 1 shows the first electrocardiogram (ECG) after successful resuscitation (Fig. 1B) and S-ICD device interrogation after resuscitation (Fig. 1A).

Two other patients developed indication for cardiac resynchronization therapy about 3 years after S-ICD implantation due to progressive heart failure and left bundle branch block without ECG findings suggestive for or symptoms of bradycardia.

## 3. Data from S-ICD Registries and Trials

Most available data rely on large observational studies analyzing the safety and efficacy of the subcutaneous ICD system like EFFORTLESS [8] and the IDE trial [9]. Burke *et al*. [10] performed a pooled 2-year follow-up analysis of both studies reporting overall three (0.3%) out of the included 882 patients who needed explantation of their S-ICD for a transvenous system for newly developed pacing indications. This correlates to results from the PRAETORIAN trial where out of the 876 enrolled patients a total number of 5 (0.5%) patients had to undergo transvenous pacemaker implantation for treatment of bradycardia [11]. Six patients (0.6%) crossed over from the subcutaneous group to a transvenous system because of required ATP, and 16 (3.5%) needed CRT during a mean follow-up of 4 years [11]. Besides, more recently the UNTOUCHED trial reported that 4 out of 1116 patients required pacing during a mean follow-up of 18 months with two of these either needing an upgrade to CRT or ATP. No patient required bradycardia-associated pacing [5]. An overview of large trials with >100 patients reporting on adverse events due to bradycardia in S-ICD patients is given in Table 2 (Ref. [4, 5, 10, 11, 12]).

**Table 2.** **Results of large trials and registries concerning need for antibradycardia pacing in S-ICD patients including more than 100 S-ICD patients reporting on rate of antibradycardia pacing needs during follow-up**.

Authors, year of publication	Number of patients included, % male, mean age	Mean follow-up duration (months)	Need for antibradycardia pacing (total number, %)
Burke *et al*. 2020 (PAS) [10]	1637, 68.5%, 53 yrs.	12	2 (0.1%)
Knops *et al*. 2020 (PRAETORIAN) [11]	426, 79.1%, 63 yrs.	49.1	4 (0.9%)
Gold *et al*. 2020 (Untouched) [5]	1111, 74.6%, 56 yrs.	18	0 (0%)
Willy *et al*. 2021 [4]	398, 67.3%, 43 yrs.	34.9	2 (0.5%)
Brouwer *et al*. 2016 [12]	140, 60%, 41 yrs.	120	2 (1,3%)
Pooled Analysis	3712, 71.1%, 53.4 yrs.	24.6	10 (0.3%)

In summary, very few patients from these trials (especially the well-designed randomized and controlled PRAETORIAN trial) had occurrence of relevant or clinically symptomatic bradycardia requiring pacemaker implantation or change to a transvenous ICD, respectively. With regard to the low frequency of patients requiring ATP and the effectiveness of ATP being highly dependent on the cycle length and type of occurring VT an adequate selection of the optimal device seems very challenging [4]. Although, having an overall low frequency of pacemaker implantation in the aforementioned studies, the risk for a subsequent pacing need of any kind has to be taken into consideration during evaluation preceding the procedure.

## 4. Discussion 

To deliver a prospective European snapshot of choosing the S-ICD or a transvenous ICD system the European Heart Rhythm Association (EHRA) published a survey of 20 centres [13]. The main reasons for choosing a transvenous device were the potential need for ATP (43.2%), CRT (40%), or permanent pacing (39.6%) [13]. An S-ICD was mostly chosen in patients of young age (66.7%), possible or already experienced lead-complications (18.5%), and increased risk for device infections (7.4%). Besides, 16.7% of the responding centres based their choice on patient preference with 13% of centres also taking an active patient lifestyle in favour of the S-ICD into account [13]. Therefore, it seems that the S-ICD is increasing as the preferred implantable device in patients expected to meet the aforementioned criteria. As for our rather young patient cohort, the disadvantage of bradycardias might not be as relevant as in comparable older cohorts where incidences of symptomatic bradycardias are much more relevant. Also, one has to consider higher incidences of complications for dual chamber ICD systems. Careful evaluation before deicing on the type of system should therefore be performed to prevent unnecessary complicational risk of often young patients. Anticipating this development, the current ESC Guidelines for the management of patients with ventricular tachycardia and prevention of SCD [6] have recommended the S-ICD with a IIa indication when pacing is not required. Of note, if the patient has a difficult venous access for intravenous lead placement, the transvenous system has recently been removed, or the patient has an increased risk for infection the S-ICD is given a IIb recommendation [6], whereas the AHA/ACC/HRS Guidelines [7] give a class I recommendation for these patients. These recommendations clearly suggest that the S-ICD is regarded a valid alternative to transvenous ICDs in patients presenting with common indication of ICD placement. However, as these guidelines rely on the knowledge of potential need for any kind of pacing, they do not offer any guidance as to how this need might be anticipated, consequently the approach to adequate patient selection is executed highly individually and is therefore prone to over- or underestimation of the potential pacing need. Also, bradycardia-associated pacing is considered to add substantial proarrhythmic potential leading to pacing-induced tachycardias. Therefore, also considering alternatives as sub-threshold pacing should be considered to prevent unnecessary ICD-Shocks in patients with expected bradycardia associated pacing needs [14].

## 5. Approaches to Anticipate Future Pacing Need

Harding *et al*. [15] sought to develop an approach for appropriate patient selection using data gathered by routine follow-up visits of patients who received a transvenous ICD system to estimate what would have happened if these individuals would have received an S-ICD. Three sets of S-ICD inclusion criteria were developed based on the predominant cardiac pathology, ECG and echocardiographic findings.

These selection criteria were then applied to the aforementioned patient cohort, estimating who would have either profited from intraventricular pacing (brady-pacing, ATP or CRT) or S-ICD during follow-up. The study cohort consisted of 951 patients almost equally receiving a transvenous dual chamber ICD (42.9%) or CRT-D device (35%) in primary (47.8%) or secondary (52.2%) prevention [4]. Here, depending on the underlying heart disease, a range from 4.7% to 35.5% of transvenous ICD recipients would have also been suitable for an S-ICD according to current ESC or AHA/ACC/HRS guidelines. This range highly depended on how strict one selected the patient cohort (QRS-duration, pre-existing CRT Indication at time of implantation). This was due to the fact, that the incidence for a patient possibly needing CRT Implantation during follow-up (3.3 years) ranged from 0% to 2.3% depending on which selection model was chosen (Option A being the most restrictive, see Table 3, Ref. [15]). Another challenge was anticipating or estimating bradycardia-associated pacing needs in patients without a clear indication for it because transvenous ICD systems are capable of backup pacing. An estimate of two up to 11% during a 5-year-follow-up did in fact profit from the transvenous device and its pacing capabilities [14]. However, in most patients with an ICD programmed to a standard anti-bradycardia stimulation rate of, e.g., VVI 40 bpm, a ventricular pacing burden of 2% might be regarded as not mandatory as pacing may take place at night or during asymptomatic bradycardia in, e.g., patients with atrial fibrillation. In our real life data set as well as in even larger data sets from other registries the rate of patients developing an indication for antibradycardia pacing is even lower and reliably below 1%.

**Table 3.** **Selection criteria devised according to clinical findings and current guidelines by the ESC and AHA/ACC/HRS [15]**.

	Description
A	S-ICD used in inherited channelopathies and idiopathic ventricular fibrillation only
B	+ hypertrophic cardiomyopathy and grown-up congenital heart disease patients
C	+ primary and secondary prevention (for ventricular fibrillation only) of SCD in patients with QRS <150 ms. Approach C reflects current ESC and AHA/ACC/HRS

ESC, European Society of Cardiology; AHA, American Heart Association; ACC, American College of Cardiology; HRS, Heart Rhythm Society; S-ICD, Subcutaneous-Implantable Defibrillator; SCD, Sudden Cardiac Death.

It is already known that the need for ATP depends greatly on the type of occurring VT and also the programming of the device itself, as the occurrence of ATP does not always assure that it was needed in the first place. Having this in mind no patient in the aforementioned trial ultimately needed ATP. This of course entails the assumption that there would’ve been no excess shocks if these patients had received an S-ICD. Estimations for ATP in a primary prevention cohort of the APPRAISE-ATP trial [16] will hopefully help optimizing the selection process in everyday clinical practice. Furthermore, as leadless pacemakers are making their way into everyday clinical practice (over 100.000 implanted devices worldwide [17] the probability towards leadless pacemakers capable of ATP with unidirectional control with the S-ICD is on the horizon, which may make the concern for complications by intravenous lead placement obsolete [12, 18].

Lastly, to help determining early mortality risk after ICD implantation, Goldenberg *et al*. [19] and Bilchik *et al*. [19] developed risk scores using patient characteristics at the time of implantation like the New York Heart Association functional class, age, urea level, QRS duration, and atrial fibrillation and the Bilchick score including diabetes, chronic obstructive pulmonary disease, and chronic kidney disease status. The Bilchik Score is considered the more generalized one, having been validated in a primary prevention cohort and demonstrated to show a nonlinear but significant relationship between score and mortality in a median follow-up of 4 years. Therefore, the score was applied to the aforementioned study cohort predicting mortality risk after device implantation. Indeed, patients suitable for an S-ICD scored lower than the ones with indications for a transvenous system with additionally restrictive selection criteria further decreasing the score, suggesting a causal relationship between benefit from an S-ICD with restrictive use.

But as these two scores have only been validated in a restrictive patient cohort the applicability in clinical practice is doubtful and should be investigated further. This is especially true for mixed large cohorts like EFFORTLESS or UNTOUCHED or our S-ICD collective which demonstrated only very few patients with pacing needs during follow-up. It may therefore be of interest to analyze patients with already existing disturbances of the AV conduction system such as AV block I°, right bundle branch block or left bundle branch block at the time of S-ICD implantation. As especially in young patients with channelopathies the development of higher degree AV blocks is described [20, 21], the necessity for changing from the S-ICD to transvenous systems may be expected during longer- follow-up. This is especially interesting as this a patient cohort currently regarded as most suitable for S-ICD implantation. As a possible limitation which hampers comparability with other trials one has to mention the low number of patients with ischemic cardiomyopathy in our patient cohort. This might partially explain the lower number of bradycardias as a relevant proportion of patients from S-ICD trials have a structurally normal heart.

## 6. Conclusions

The risk of relevant bradycardia seems to be very low in large registries and trials. We were able to show that concerns regarding the S-ICD in terms of a missing pacing option are often needless if patients are well selected with no pacing indication at implantation. Nonetheless, careful balancing the advantages and disadvantages of each technology is warranted and shared decision making with the patient should be aspired.
